# Mapping the giants: a bibliometric analysis of the top 100 most-cited thyroid nodules studies

**DOI:** 10.3389/fmed.2025.1555676

**Published:** 2025-03-25

**Authors:** Xinfeng Zhou, Mingjun Liu, Tianjiao Gao, Yi Tan, Xiao Wang, Long Yang, Shengxian Xu, Rui Wang, Haoyang Gao, Shaotao Chen

**Affiliations:** ^1^Department of Acupuncture and Massage, Changchun University of Chinese Medicine, Changchun, Jilin, China; ^2^The Affiliated Hospital of Changchun University of Chinese Medicine, Changchun, Jilin, China; ^3^Department of Chinese Medicine, Changchun University of Chinese Medicine, Changchun, Jilin, China

**Keywords:** thyroid nodules, bibliometric analysis, VOSviewer, CiteSpace, bibliometrix

## Abstract

**Background:**

Thyroid disease continues to be one of the most prevalent disease groups worldwide, with its frequency and distribution being impacted by numerous factors. Significant progress has been achieved in recent years in thyroid nodules, largely due to the advent of novel detection and diagnostic techniques. This study aims to scrutinize the top 100 most frequently cited articles in thyroid nodule research, utilizing bibliometric analysis to identify trends, highlight critical focal points, and lay a groundwork for forthcoming investigations.

**Methods:**

A comprehensive literature search was carried out using the SCI-E database, and all the recorded results were downloaded in plain text format for detailed analysis. The key terms analyzed with VOSviewer 1.6.18, CiteSpace 6.3r1, bibliometrix in R Studio (v.4.4.1), and Microsoft Excel 2021 software include country, institution, author, journal, and keywords.

**Results:**

The publication timeframe extends from 1 January 2003 to 31 December 2021, reaching a peak citation count of 9,100. Notably, the United States leads in the number of published articles, with Harvard University standing out as a prestigious institution. These articles were featured in 45 diverse journals, with THYROID leading in publication volume. Nikiforov Yuri E. was the most prolific first author, appearing 10 times. Keyword analysis highlighted traditional research themes such as “fine needle aspiration,” “carcinogens,” and “management.” However, “deep learning” has surfaced as a significant area of focus in recent studies.

**Conclusion:**

This study has extracted the bibliometric characteristics of the top 100 most-cited articles pertaining to TNs, providing an invaluable reference for upcoming studies. Through meticulous analysis, it has been determined that the primary research concentrations encompass the diagnosis of benign or malignant TNs, the management of TNs, and the subsequent monitoring of TNs, with deep learning emerging as a pivotal area of exploration.

## 1 Introduction

The thyroid gland, situated just below the thyroid cartilage at the front of the neck, serves as a pivotal endocrine organ in the human body. Its main function is to secrete thyroid hormone, a crucial component in regulating the body’s metabolism. Thyroid nodules (TNs) represent the most common thyroid disorders. Some TNs display well-defined borders, whereas others show irregular shapes. Nodular thyroid disease presents in diverse forms, such as solitary nodules, multinodular goiter, nodular goiter stemming from autoimmune goiter, and inaccessible thyroid nodules. Typically, TNs appear as solid nodules, cystic nodules, or a combination of both. TNs are widespread, with up to 65% of the general population affected (1). Approximately 4 to 7% of the population experiences palpable TNs, and the occurrence of TNs increases with age (2). The prevalence of TNs appears to be higher among women, potentially influenced by factors such as age, radiation exposure, and elevated levels of goitrogenic substances in the diet. Fortunately, recent advancements in the field of TNs, driven by the introduction of new detection and diagnostic techniques, have led to a remarkable improvement in the detection rate of TNs. However, this progress has also been accompanied by a rise in the incidence of thyroid cancer (3). TNs are extremely important in clinical settings because they have the potential to cause thyroid dysfunction, occasional compression symptoms, and the need to rule out the possibility of thyroid cancer (4).

There is an abundance of articles discussing TNs, and although these medical publications cover crucial topics in the field, it remains a challenge for researchers to pinpoint the most influential articles, research trends, and future directions. Bibliometric analysis emerges as an invaluable tool, primarily relying on the statistical assessment of published articles or books to discern trends and keep pace with the latest advancements. Notably, bibliometrics has found extensive application in the field of medicine, encompassing studies on TNs (5–7). However, performing a comprehensive bibliometric analysis on the most-cited articles in the field of TNs will significantly aid in understanding the discipline’s future developmental trajectory. Therefore, the goal of this study is to objectively describe the top 100 most-cited TN articles utilizing CiteSpace and VOSviewer. Furthermore, the study aims to scrutinize their bibliometric characteristics, facilitating the discovery of prevalent research trends and possible future directions in this field.

## 2 Materials and methods

### 2.1 Search strategy

**Figure 1:**
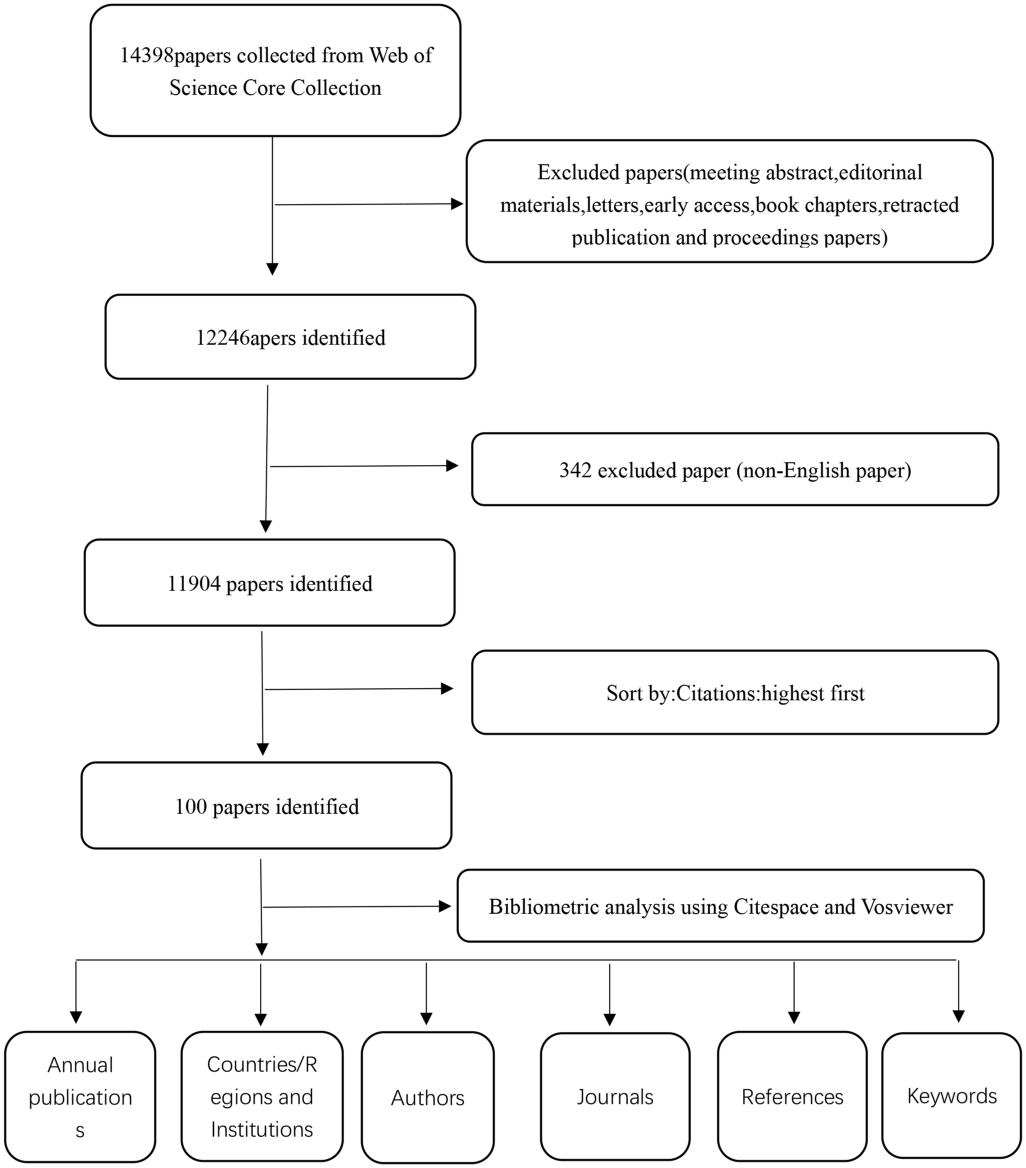
The figure presents a one-frame flow chart that illustrates the comprehensive criteria and the step-by-step process of conducting a bibliometric analysis on publications chosen from the WOS database, specifically exploring the thyroid nodules.

An extensive search was carried out on 14 August 2024, within the core collection of Web of Science, employing the following public notices: TS = (“Thyroid Nodule (All Fields)” OR “Nodules, Thyroid (All Fields)” OR “Nodule, Thyroid (All Fields)” OR “Thyroid Nodules (All Fields)”). After obtaining the search results, article and review types were selected, while conference abstracts, preemptive experience articles, edited materials, letters, collections, anthologies, news projects, book chapters, hardware reviews, and withdrawn publications were excluded. Only articles written exclusively in English were selected, excluding any use of German, French, Spanish, Portuguese, Korean, Hungarian, Italian, Chinese, Russian, Serbian, Turkish, Polish, Greek, Icelandic, Lithuanian, or any other unspecified languages. A total of 11,904 articles underwent rigorous screening and were subsequently excluded. Please refer to Figure 1 for detailed information regarding the literature extraction process.

### 2.2 Data collection

The retrieved literature was organized according to the number of citations, arranged in descending order, and the 100 most frequently cited articles were selected for further examination. To eliminate duplicates or excessive citations, two reviewers rigorously screened the chosen articles. The exported content is displayed in a plain text format, with data delimited by tabs. This record offers a thorough listing of referenced citations, including the author, country, institution, title, publication year, citation, journal, and other relevant details.

### 2.3 Bibliometric analysis

For bibliometric and knowledge mapping analysis, we used CiteSpace 6.3r1, VOSviewer 1.6.18, bibliometrix in RStudio (version 4.4.1), and Microsoft Excel 2021.

CiteSpace is a software developed by Professor Chao Mei Chen from Drexel University located in Philadelphia, Pennsylvania, United States. It was specifically crafted to visualize various knowledge domains (8, 9). In this study, the time frame has been adjusted to cover the period from 2003 to 2021, with the parameter threshold set at 1, Top N (where *N* = 25), LRF = 2.5, LBY = 5, and e = 1.0. The implemented operations include the analysis of countries, institutions, keywords, and bursts of keyword activity.

The dataset underwent fundamental statistical analysis using RStudio (v.4.4.1). The data were stored in bibliometrix format, and the biblioshiny package was utilized to extract diverse features related to research literature from 2003 to 2021. These features encompassed crucial information, including prominent authors, trends in author output, and the most frequently cited global studies, forming the foundation for quantitative assessment.

VOSviewer, created by Eck and Waltman from the Leiden Scientific and Technological Research Center in the Netherlands, is a software specifically crafted for mapping scientific knowledge. This software can establish and visualize connections between network data, revealing the structure, progression, and collaboration within the realm of knowledge. Its standout attributes include exceptional graphical visualization and proficiency in handling large-scale data analysis. It is utilized to accomplish tasks such as accessing journals and identifying authors referenced in this article.

This article employs Microsoft Excel 2021 to investigate yearly publication trends, concurrently exploring the impact index of magazines and periodicals on the Web of Science in 2023.

## 3 Results

### 3.1 Publication trends

**Figure 2:**
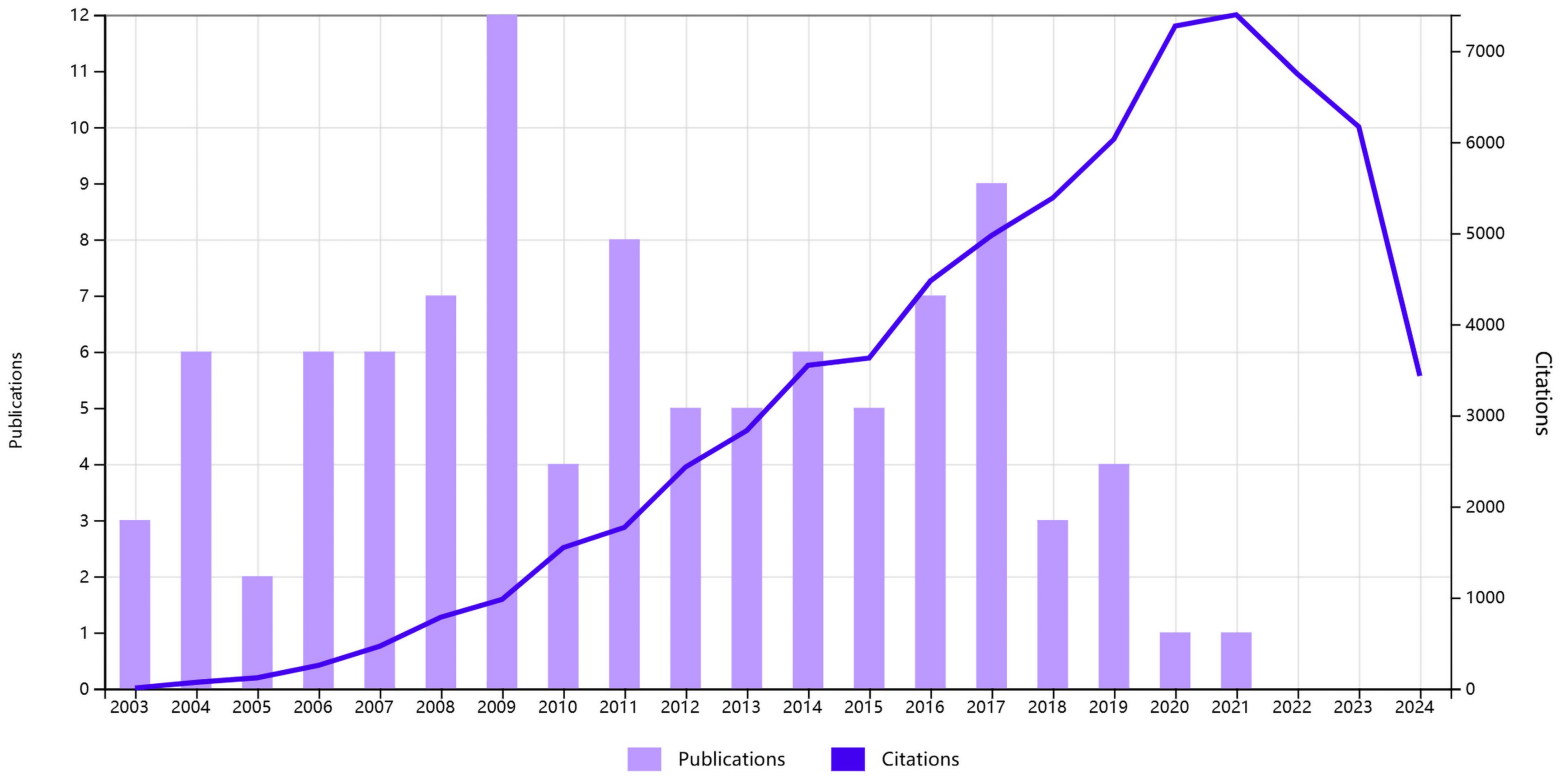
Distribution trend from January 1, 2003, to December 31, 2021.

All 100 of the most highly cited studies, from 2003 to 2021 (refer to Figure 2), revealed 2009 as the most productive year with 12 publications. Among these top 100 articles, 77 were original research articles (77%) and 23 were reviews (23%). Table 1 highlights the top 10 most-cited articles. Notably, within this elite group, the most frequently cited article was authored by Bryan R. Haugen and his team in the THYROID journal in 2016 (10), garnered an exceptional 9,100 citations, setting it apart as the most influential. On the other hand, the least cited article had 242 citations. The average citation count per article stood at 720.7. Furthermore, 41 articles surpassed 500 citations, while every article in the list achieved at least 100 citations.

**Table 1:** Top 10 cited articles related to thyroid nodules.

Rank	Title	Author	Journal	Total citations	Year
1	2015 American Thyroid Association Management Guidelines for Adult Patients with Thyroid Nodules and Differentiated Thyroid Cancer The American Thyroid Association Guidelines Task Force on Thyroid Nodules and Differentiated Thyroid Cancer	Haugen, Bryan R	Thyroid	9,100	2016
2	Revised American Thyroid Association Management Guidelines for Patients with Thyroid Nodules and Differentiated Thyroid Cancer	Cooper, David S.	Thyroid	5,130	2009
3	International Association for the Study of Lung Cancer/American Thoracic Society/European Respiratory Society International Multidisciplinary Classification of Lung Adenocarcinoma	Travis, William D	Journal of Thoracic Oncology	3,559	2011
4	Increasing incidence of thyroid cancer in the United States, 1973–2002	Davies, L	JAMA-Journal of the American Medical Association	2,613	2006
5	Integrated Genomic Characterization of Papillary Thyroid Carcinoma	Agrawal, Nishant	Cell	2000	2014
6	The Bethesda System for Reporting Thyroid Cytopathology	Cibas, Edmund S	American Journal of Clinical Pathology	1886	2009
7	The Bethesda System for Reporting Thyroid Cytopathology	Cibas, Edmund S	Thyroid	1886	2009
8	Management guidelines for patients with thyroid nodules and differentiated thyroid cancer	Smith, BR	Thyroid	1,589	2006
9	Trends in Thyroid Cancer Incidence and Mortality in the United States, 1974–2013	Lim, Hyeyeun	JAMA-Journal of the American Medical Association	1,438	2017
10	2017 Guidelines of the American Thyroid Association for the Diagnosis and Management of Thyroid Disease During Pregnancy and the Postpartum	Alexander, Erik K	Thyroid	1,425	2017

### 3.2 Distribution of countries/regions

**Figure 3:**
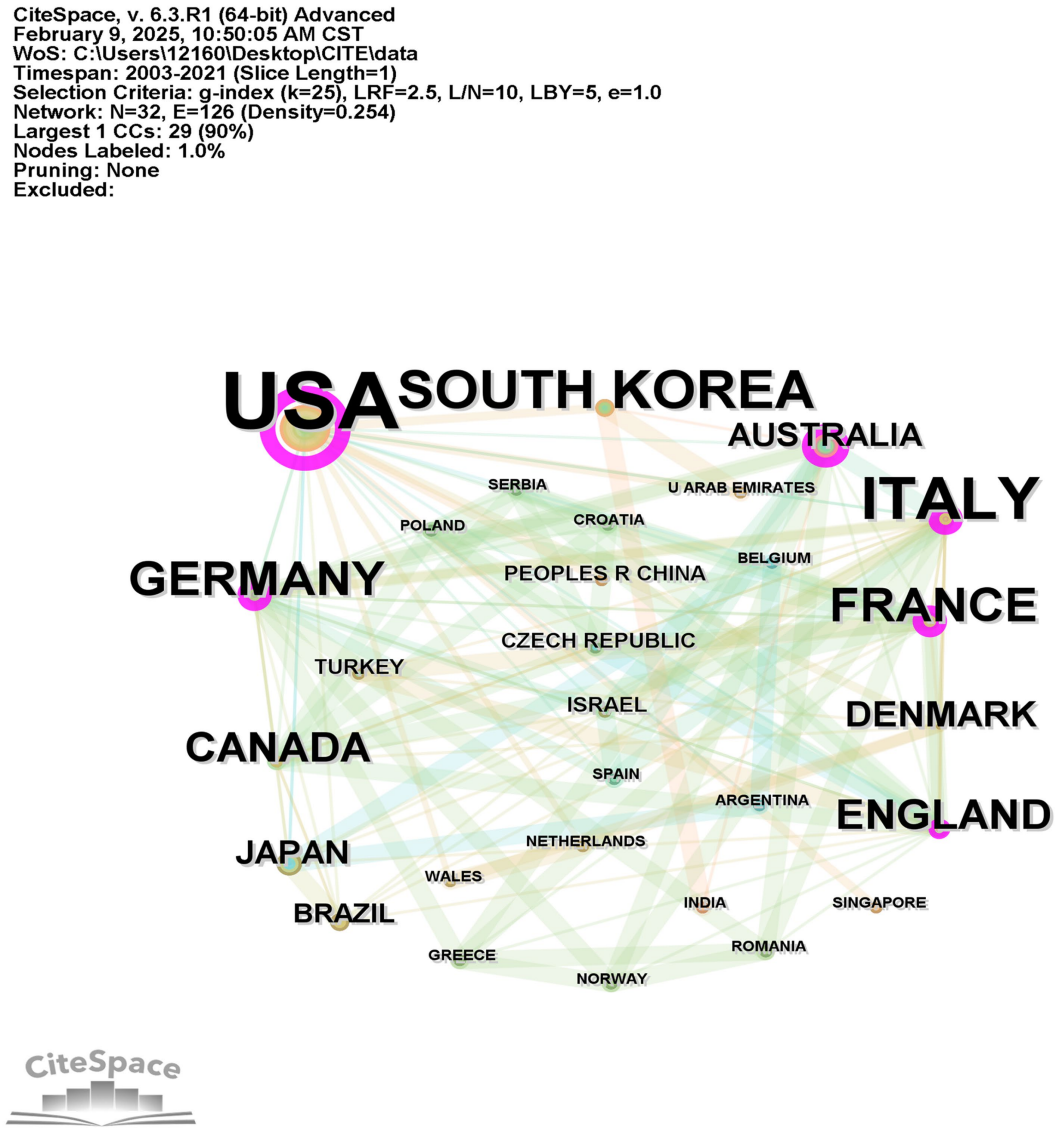
A visualization map of countries/regions.

The 100 articles with the highest citation counts span 116 countries/regions. Table 2 lists the top 10 most-cited countries/regions. In terms of publications, the United States (*n* = 61), Italy (*n* = 20), and South Korea (*n* = 13) stand at the forefront. The United States tops the list with a centrality of 0.83, followed by South Korea at 0.22 and the United Kingdom (UK) at 0.16. Notably, the United States excels in publication count, citations, and centrality. Figure 3 illustrates the collaborative network among countries using a national co-occurrence map. Here, nodes represent countries, with their size reflecting the degree of national circulation. The largest circle, representing the United States, underscores its significant influence in the region. The purple segment highlights the center, occupied by the United States, showcasing its extensive collaboration with other nations.

**Table 2:** Top 10 countries/regions related to thyroid nodules.

Rank	Country	Publications	Centrality	H-index	Citations per papers
1	USA	61	0.83	61	882.95
2	Italy	20	0.1	20	1188.7
3	South Korea	13	0.22	13	408
4	Germany	10	0.14	10	789.5
5	France	9	0.1	9	2066.67
6	Canada	7	0.06	7	1872
7	England	7	0.16	7	730.71
8	Denmark	5	0.05	5	916.2
9	Japan	4	0.05	4	688.75
10	Brazil	3	0	3	920.67

### 3.3 University and institutional

**Figure 4:**
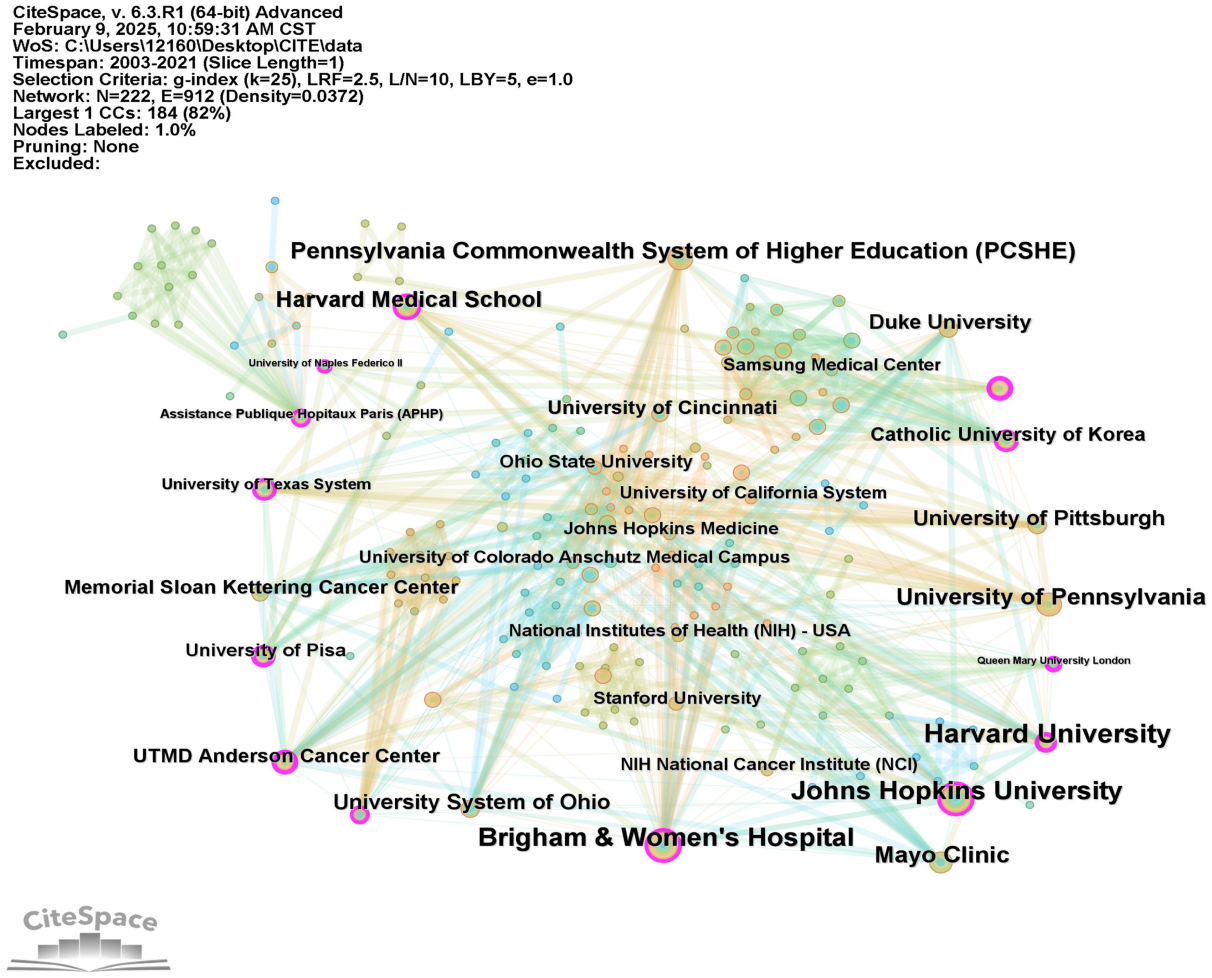
A visualization map of institutions.

A total of 605 institutions participated in the research on this topic. The results from network pruning (refer to Figure 4) revealed a total of 222 nodes and 912 connections. Table 3 lists the top 10 institutions ranked by their literature output. Harvard University led the pack with 17 publications, followed closely by Johns Hopkins University and Brigham and Women’s Hospital, both with 14 publications. Notably, all institutions in the top 10 are from the United States, highlighting the country’s extensive exploration and solid scientific research foundation in this field. Harvard University’s impressive h-index underscores its significant impact on research and publications related to this topic.

**Table 3:** Top 10 institutions related to thyroid nodules.

Rank	Institution	Count	Country	H-index	Citations per papers
1	Harvard University	17	USA	17	1229.35
2	Johns Hopkins University	14	USA	15	1000.2
3	Brigham & Women’s Hospital	14	USA	14	1399.21
4	University of Pennsylvania	12	USA	15	1591.27
5	Pennsylvania Commonwealth System of Higher Education (PCSHE)	12	USA	13	1101.31
6	Mayo Clinic	11	USA	11	1905.73
7	Harvard Medical School	10	USA	10	1633.1
8	University System of Ohio	9	USA	11	1788.55
9	University of Pittsburgh	9	USA	13	1101.31
10	Duke University	8	USA	8	1777

### 3.4 Journals

**Figure 5:**
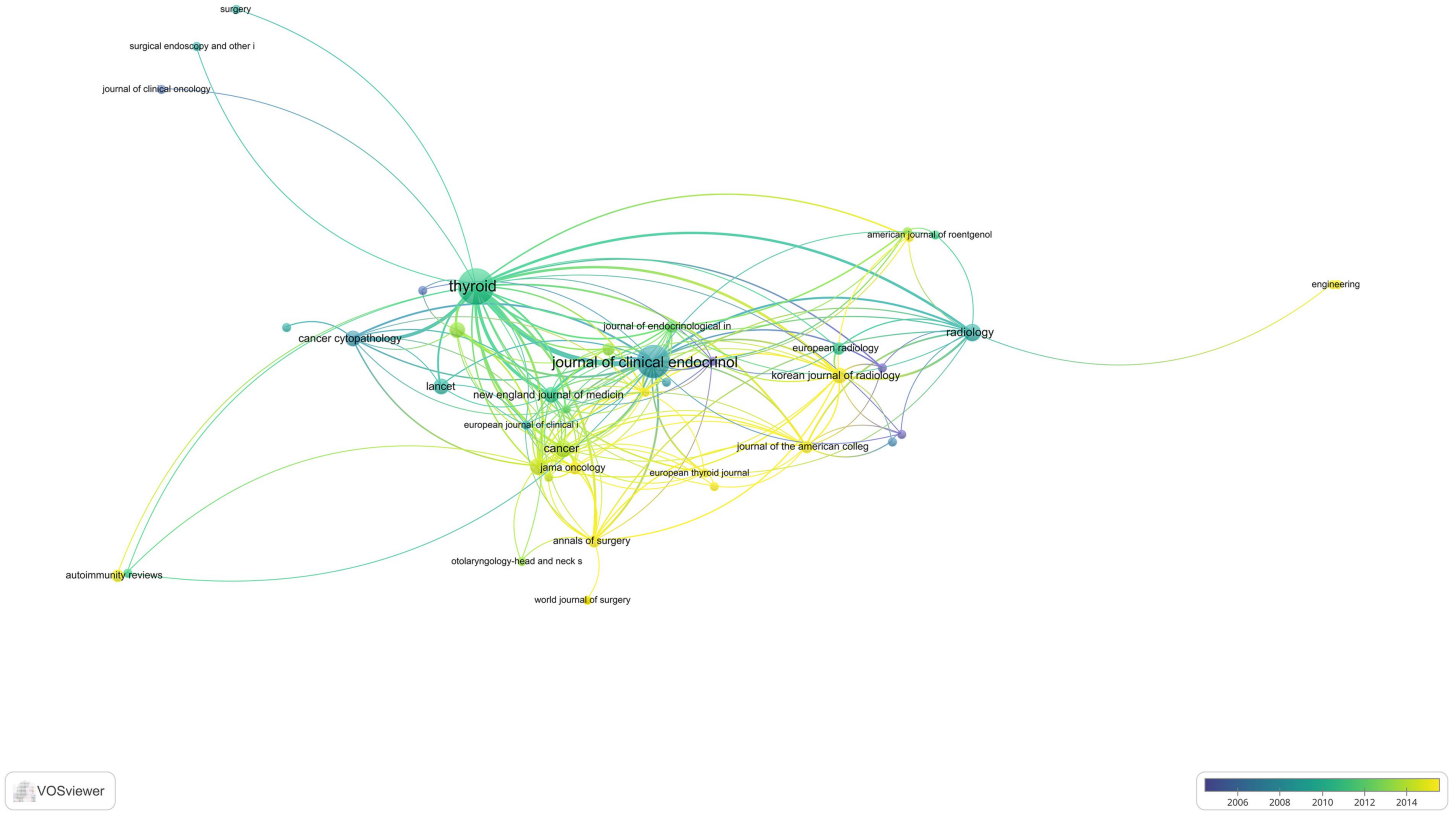
A visualization map of Journal.

The top 100 most frequently cited articles on thyroid nodules hail from 45 journals, and Table 4 highlights journals that have featured three or more of these articles. Notably, THYROID stands out with a total of 17 publications, indicating its significant role in the field. The Journal of Clinical Endocrinology and Metabolism also shines with 14 articles. Interestingly, the majority of the journals listed primarily belong to Division 1 or a higher rank of the Chinese Academy of Sciences. However, a striking disparity exists as half of these journals possess an impact factor of less than 10, reflecting an imbalance in thyroid nodule research quality. Although recognized by esteemed journals, this observation underscores the need for researchers to maintain meticulous research design, prioritize quality assurance, foster international collaborations, undertake large-scale multicenter clinical studies, and aim to publish articles with high impact. Seven journals from the United States echo the findings of various nations and institutions, and their coexistence is visually represented in Figure 5.

**Table 4:** Journals that publish 3 or more articles on thyroid nodules.

Rank	Journal	Count	IF (2023)#	JCR (2023)	Country	Average citations
1	Thyroid	17	5.8	Q1	USA	1376.7059
2	Journal of Clinical Endocrinology & Metabolism	14	5	Q1	USA	456.1429
3	Cancer	4	6.1	Q1	USA	321.5
4	JAMA-Journal of the American Medical Association	4	63.1	Q1	USA	1193.5
5	Radiology	4	12.1	Q1	USA	584.5
6	Cancer Cytopathology	3	2.6	Q3	USA	422.3333
7	Korean Journal of Radiology	3	4.4	Q1	South Korea	459.6667
8	Lancet	3	98.4	Q1	England	548
9	Nature Reviews Endocrinology	3	31	Q1	England	543.3333
10	New England Journal of Medicine	3	96.2	Q1	USA	817.6667

### 3.5 Authors

**Figure 6:**
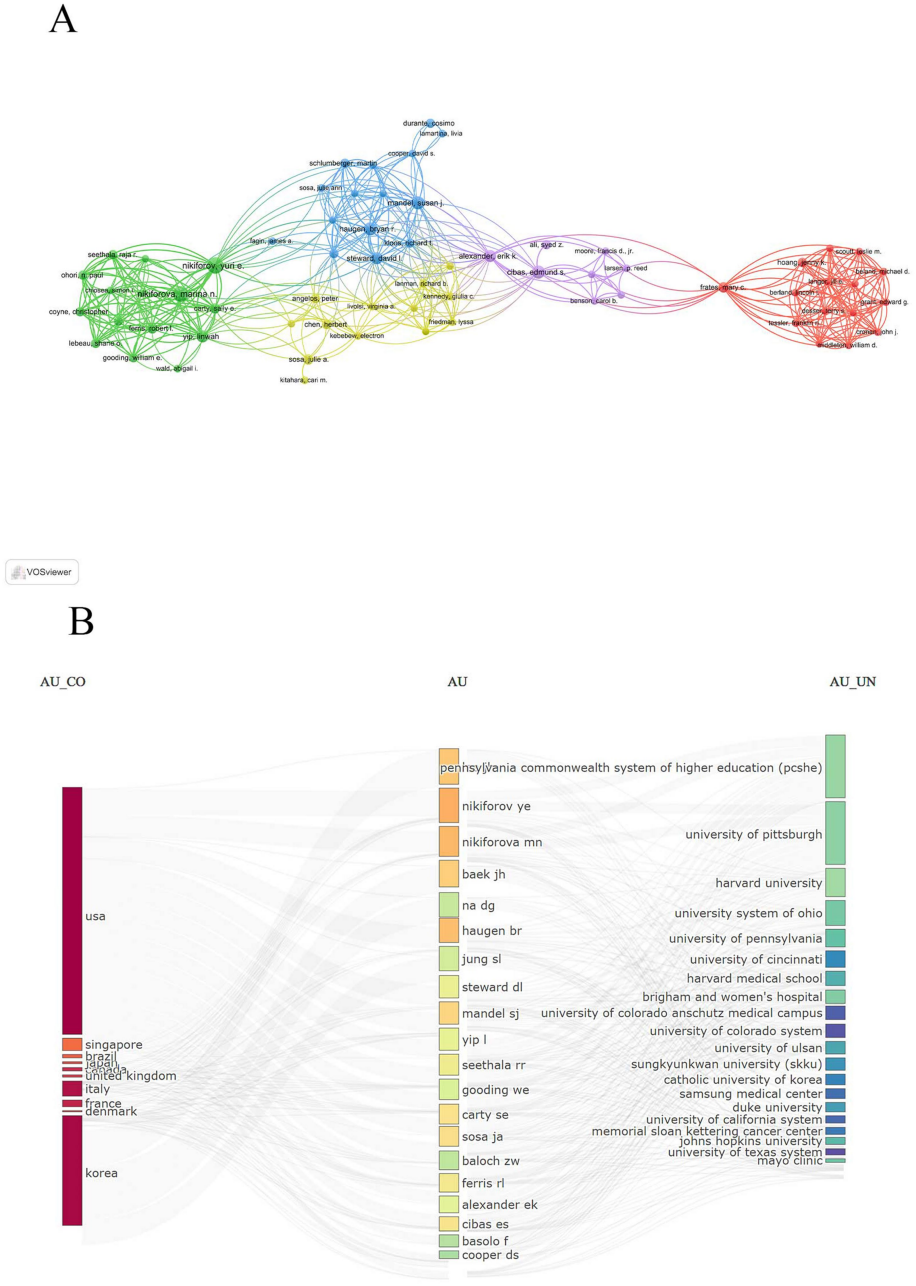
A visualization map of Author. **(A)** Author’s co-occurrence chart. **(B)** Chart showing the relationship between countries, authors and institutions.

We analyzed the author collaboration network within the 100 most highly cited TN literature, utilizing data from the Web of Science Core Collection (WOSCC) database. This comprehensive study encompassed a total of 605 authors. Referring to Table 5, we observe that Nikiforov Yuri E. is the most prolific author, with a total of 10 publications. On the other hand, Susan J Mandel stands out as the author with the highest citation count, accumulating an impressive 17,446 citations. Additionally, Mandel Susan J. holds the distinction of having the highest average citations. Figure 6A visually represents the co-occurrence of authors, where authors shaded in the same color denote a stronger collaboration frequency. Evidently, among the high-yield authors, Nikiforov Yuri, Nikiforova Marina, and Yip Linwah demonstrate active collaboration. Moreover, clusters are formed by Haugen Bryan R., Mandel Susan J., Steward David L., Cibas Edmund S., and Alexander Erik K., indicating a tighter collaboration among these high-yielding authors. Moving on to Figure 6B, it elucidates the intricate relationships between countries, authors, and institutions. This figure underscores the significant influence authors from the United States have had in shaping this field.

**Table 5:** Authors of more than 5 publications on thyroid nodules.

Rank	Author	Publications	Citations	Average citations
1	Nikiforov, Yuri E	10	12,841	1284.1
2	Nikiforova, Marina N	9	3,741	415.6667
3	Cibas, Edmund S	6	6,398	1066.3333
4	Haugen, Bryan R	6	16,383	2730.5
5	Mandel, Susan J	6	17,446	2907.6667
6	Alexander, Erik K	5	12,302	2460.4
7	Steward, David L	5	15,817	3163.4
8	Yip, Linwah	5	1708	341.6

### 3.6 Keywords and keywords burst

**Figure 7:**
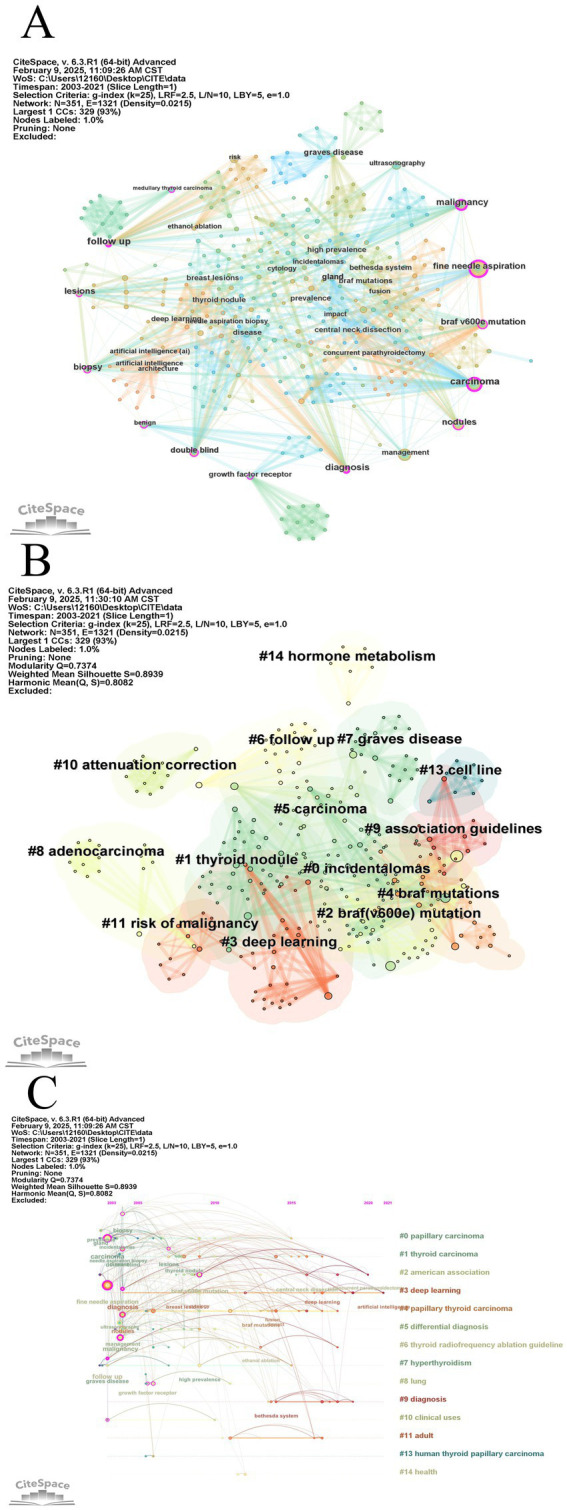
A visualization map of keywords. **(A)** A visualization map of keywords. **(B)** CiteSpace visualization timeline view of keywords clustering analysis related to thyroid nodules. **(C)** CiteSpace visual timeline view of keyword clustering analysis related to thyroid nodules.

We used CiteSpace to generate a visual representation of the frequently occurring keywords in the literature related to this study’s topic. Figure 7A displays this visualization, where larger circles represent higher frequencies of corresponding keywords in the literature. Ten keywords appear more than 10 times, and Table 6 lists the 10 most popular keywords ranked by their frequency. Notably, the top keywords include “fine needle aspiration,” “carcinoma,” and “management.” The majority of the top keywords focus primarily on 2004. In CiteSpace, keywords with high centrality (centrality ≥0.01) can be easily identified as pivotal points on the keyword frequency knowledge map, signifying prevalent research hotspots in the field. Specifically, “fine needle aspiration” has a centrality of 0.35, “follow-up” has a centrality of 0.32, and “carcinoma” has a centrality of 0.30, highlighting their key roles in the research network.

**Table 6:** The top 10 most frequent keywords on thyroid nodules.

Rank	Keywords	Counts	Centrality	Year
1	Fine needle aspiration	44	0.35	2003
2	Carcinoma	26	0.30	2003
3	Management	24	0.07	2004
4	Malignancy	17	0.24	2004
5	Nodules	16	0.15	2004
6	Diagnosis	15	0.28	2004
7	Ultrasonography	15	0.04	2004
8	Biopsy	14	0.18	2004
9	Lesions	10	0.11	2007
10	Follow up	10	0.32	2003

In citation space, the log-likelihood ratio (LLR) algorithm was used to cluster keywords derived from both Chinese and English literature. As the clustering process groups more nodes and research hotspots together, the total number of clusters decreases. The clustering effectiveness of CiteSpace can be gauged using two key metrics: the module value (Q) and the average contour value (S). A Q value exceeding 0.3 signifies a notable divided community structure, while an S value above 0.5 suggests reasonable clustering. When the S value hits 0.7, the clustering efficiency is considered highly effective and compelling. Figure 7B displays a keyword clustering map consisting of 351 nodes and 1,321 connections, with a Q of 0.7374 (> 0.3) and an S of 0.8082 (> 0.7). These figures underscore the efficiency and rationality of the clustering, implying high reliability in the results. Notably, a smaller cluster number corresponds to a broader range of encompassed keywords. The figure predominantly showcases 14 primary clusters, including #0(incidentalomas), #1(thyroid nodule), #2(braf v600e mutations), #3(deep learning), #4(braf mutations), #5(carcinoma), #6(follow up), #7(Graves’ disease), #8(adenocarcinoma), #9(association guidelines), #10(attenuation correction), #11(risk of malignancy), #13(cell line), and #14(hormone metabolism).

Figure 7C presents a chronological perspective of keyword clustering analysis, featuring a timeline view. On the right side of the figure, brightly colored horizontal lines represent various clusters, each corresponding to a distinct set of keywords. Nodes, positioned along these lines, symbolize individual keywords, and their spatial placement along the horizontal axis indicates the year of their first appearance in academic literature. This offers a comprehensive temporal representation of the evolution of keyword clusters. The largest cluster corresponds to “papillary carcinoma,” followed by “thyroid carcinoma,” “American Association,” “deep learning,” and several others.

**Figure 8:**
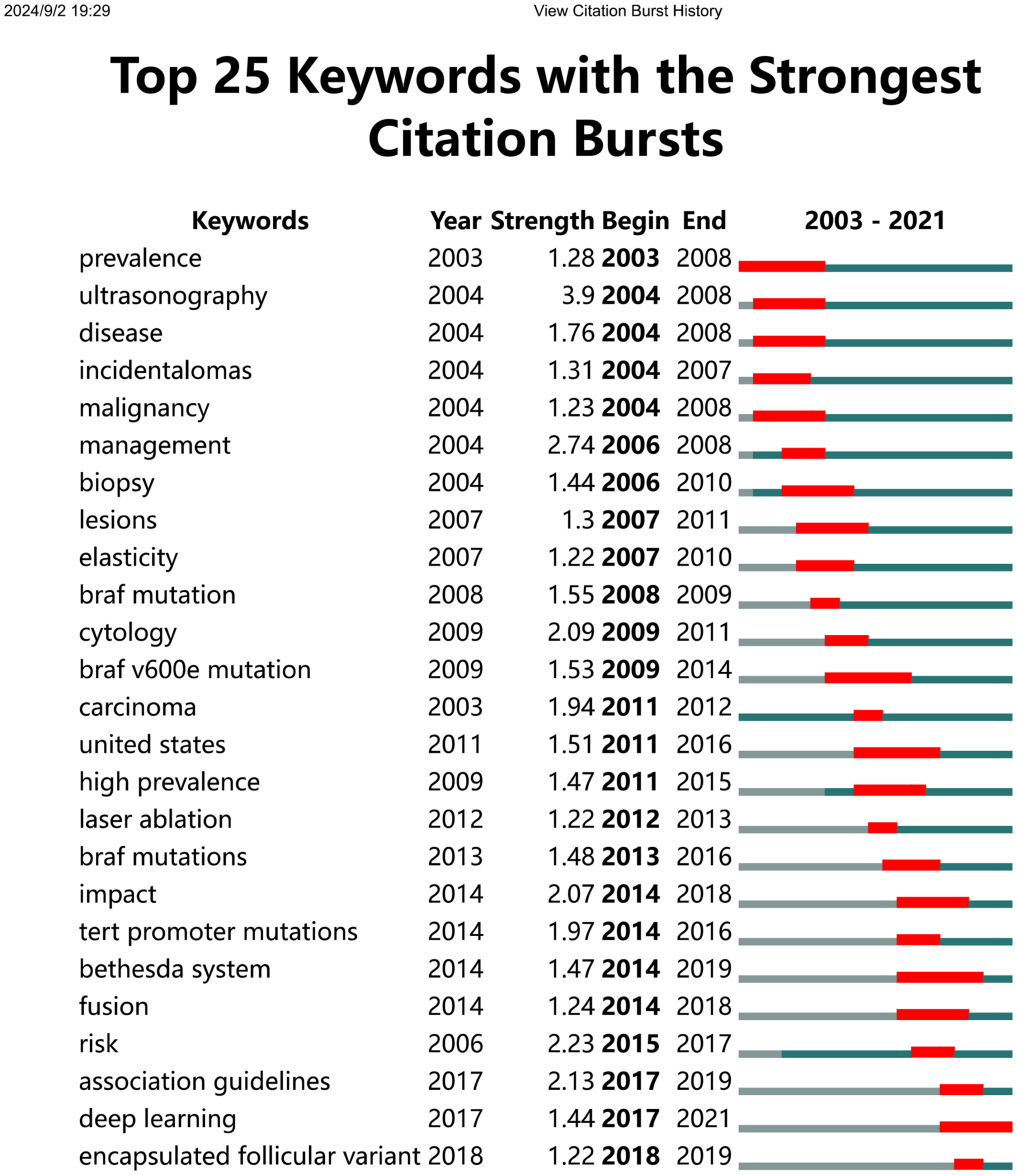
Top 25 keywords with the strongest citation bursts involved in thyroid nodules.

The emergence of keywords refers to the sudden and significant increase in the use of specific keywords within their respective fields over a particular period. Figure 8 showcases the top 25 keywords that have undergone the most notable citation surges. Among them, “ultrasonography” stands out as the most prominent, while “management,” “risk,” “prevalence,” “braf v600e mutation,” “united states,” and “bethesda system” have experienced the longest-lasting citation spikes. It is worth mentioning that “deep learning” continues to exhibit explosive growth until 2021.

## 4 Discussion

### 4.1 General information discussion

In this study, we conducted a bibliometric analysis of the 100 most influential publications in the domain of TNs. Through this approach, we were able to delve into the knowledge base, key focal points, and cutting-edge aspects pertaining to TNs. This provided us with vital quantitative insights into both longstanding research and recent progress in the field, significantly deepening our understanding of TNs. Furthermore, we analyzed trends and hotspots in TN research, which we anticipate will open up new avenues for future investigations into TN therapies.

Upon examination of Figure 2, the most notable research achievement occurred in 2009, evidenced by the record-high number of publications. This positive trend may have originated from the release of essential guidelines, progress in diagnostic technology, the promotion of epidemiological studies, the need for clinical management standardization, and the increase in multidisciplinary research. Generally speaking, the accrual of citations for articles is a gradual process. Scientific works typically receive their initial citation within 1–2 years after publication, ultimately peaking around a decade later. Consequently, recently released articles may not have accumulated sufficient citations to be included among the top 100, whereas older articles may possess a higher citation tally. The articles highlighted in our study possess the prospect of attracting greater scholarly attention in the forthcoming years.

The United States holds the distinction of being the foremost contributor in this field, with its publication count, centrality, and h-index securely atop the list. This dominance reflects a deep-seated passion for relevant research, a consistent output of high-quality work, and an overall exceptional level of inquiry. As a result, the United States has laid a strong groundwork for exploring this topic, providing an invaluable resource for scholars in the field. Notably, 90% of the top 10 contributing countries are considered developed, highlighting the relatively weaker foundation and minimal influence of developing countries in this research area. Hence, it is crucial for developing nations to consistently strengthen their partnerships with developed countries, aiming to enhance their scientific research capabilities. Harvard University stands out as the institution that has made the most notable contribution, indicating its commitment to conducting top-notch research in the domain of TNs. Its global recognition underscores the scientific rigor and professionalism inherent in its research on this subject. When ranked by their academic output, universities account for 80% of the top 10 institutions, emphasizing their pivotal role in advancing research on this topic. The majority of articles focus on thyroid-related issues, showcasing the latest advancements across multiple fields, ranging from primary healthcare to clinical applications. Moreover, it provides the only comprehensive and updated source for the American Thyroid Association’s (ATA) guidelines on thyroid disease management. Prestigious medical journals such as JAMA—Journal of the American Medical Association, The Lancet, and the New England Journal of Medicine are among those that have published more than three articles on this topic. The text demonstrates that research on this topic has gained international recognition, boasting high credibility and awareness. This recognition lays a solid foundation for future investigations on thyroid nodules (TNs). Nikiforov Yuri E. emerges as the most prolific author, whose research predominantly focuses on the pathological characteristics, diagnostic criteria, treatment principles, and developmental changes of thyroid carcinoma. His work also encompasses histological, immunohistochemical, and molecular investigations (11–15). Among authors who have published over five articles on this subject, a consistent group has emerged, with core authors offering valuable direction and guidance to future readers interested in TNs. Notably, research on TNs in the United States has yielded exceptional results, potentially attributed to its economic and technological advancements. Our comprehensive analysis, encompassing institutions, journals, and authors, ensures accuracy.

The article with the highest citation rate is a study led by Bryan R Haugen and his team, which was published in Thyroid in 2016 (10). The guideline provides a comprehensive overview of the entire process involved in diagnosing, treating, and monitoring TNS and differentiated thyroid cancer. It presents clinicians with detailed, evidence-based recommendations essential for standardizing clinical practices and reducing treatment variations and inconsistencies. The guideline highlights the importance of ultrasonography in evaluating TNS and points to a potential correlation between specific ultrasound characteristics and the risk of malignancy. Additionally, it standardizes the indications, procedures, and interpretations of fine needle aspiration (FNA) biopsy results, improving diagnostic accuracy and consistency. Clear guidance is given on key aspects, including the surgical scope for thyroid cancer treatment, indications and dosages for radioactive iodine therapy, and the level of thyroid hormone inhibition therapy required. During the development of the guidelines, we systematically collected, evaluated, and analyzed various relevant clinical research evidence, such as randomized controlled trials, cohort studies, case–control studies, and others. Through rigorous grading of the evidence, we guaranteed the scientific rigor and reliability of our recommendations. The interdisciplinary cooperation model, spearheaded by a multidisciplinary expert team convened by the American Thyroid Association, facilitates the amalgamation of diverse expertise and experience, enhancing the comprehensiveness of the guidelines. Developed by the esteemed American Thyroid Association, a foremost professional organization in thyroid diseases, the guidelines are universally recognized for their professionalism and authority. This comprehensive and practical guideline provides clinicians with a thorough diagnosis and treatment process, covering everything from the initial patient consultation to long-term follow-up. It is highly instructive and can be easily applied in real-world clinical settings. Additionally, this guideline is not static; it will be regularly updated and revised to reflect the latest research findings and advancements in clinical practice. This dynamic approach ensures that the guidelines always align with the most recent medical knowledge and technology, maintaining their relevance and usefulness in guiding clinical decision-making.

In 2009, the articles published by the American Thyroid Association’s (ATA) Guidelines Taskforce on Thyroid Nodules and Differentiated Thyroid Cancer achieved the second-highest citation rate (16) on THYROID. The guideline showcases the initial systematic integration of the diagnosis and treatment process for TNs and differentiated thyroid cancer. It provides evidence-based suggestions covering the entire process, from initial assessment and interpretation of pathological findings to extended postoperative care. Many recent studies have been converted into practical clinical guidance using a strict evidence grading system. Additionally, discussions on ultrasound technology, molecular testing, and dynamic risk stratification are incorporated, expanding on the previous guidelines set in 2006 and laying the groundwork for further enhancing future guidelines. The literature preceding 2008 underwent a comprehensive review, and the quality of research and the strength of recommendations were evaluated using the revised American Preventive Services Task Force evidence grading system. This grading system was collaboratively crafted by leading experts in endocrinology, surgery, nuclear medicine, and various other fields, ensuring the provision of thorough recommendations. To keep the guidelines current and relevant, new evidence is continuously assessed. These guidelines have become the benchmark for thyroid disease management worldwide, acting as a foundation for subsequent multinational guidelines and significantly narrowing diagnostic and therapeutic inconsistencies. Debatable issues, such as the pros and cons of prophylactic lymph node dissection and the appropriateness of radioiodine therapy for patients at low risk, have sparked numerous follow-up studies. By mitigating overtreatment, these guidelines have contributed to reducing the risks of complications, including recurrent laryngeal nerve injury and hypoparathyroidism, thereby optimizing the tailored approach to TSH inhibition therapy. The guidelines have been seamlessly integrated into medical education curricula and play a pivotal role in shaping health insurance policies. Additionally, its evidence grading system provides a valuable reference for guideline development across various domains. The primary contribution of the study lies in converting complex clinical evidence into a practical diagnosis and treatment pathway, ensuring a careful balance between therapeutic effectiveness and patient safety. Its impact extends beyond standardizing clinical practices, fostering deeper exploration of thyroid disease research through consistent updates.

### 4.2 Hot topics and introductory discussion

FNA biopsy entails puncturing the target nodule directly with a suitably sized needle to aspirate cells for pathological examination. Additionally, thyroid fine needle aspiration biopsy can also be used for specific benign thyroid nodules (17). The diagnosis of TNs mainly depends on ultrasound-guided FNA, which has become the foundation of diagnosis owing to its precision, sensitivity, specificity, reproducibility, and cost-efficiency. Its popularity among clinicians and patients alike stems from its multiple benefits, such as reduced tissue damage, minimized patient discomfort, a straightforward surgical process, and fewer complications. Studies have shown that for nodules with a maximum diameter of less than 1 cm, the accuracy of FNA ranges from 60 to 94%. For nodules with a maximum diameter exceeding 4 cm, the accuracy lies between 80.3 and 87.5% (18). FNA, strongly recommended in several guidelines, provides a relatively accurate preoperative diagnosis to determine the nature of thyroid nodules by categorizing thyroid lesions into six Bethesda groups (19). As a diagnostic tool, ultrasound-guided FNA has proven to be highly precise for thyroid nodules, significantly reducing the requirement for unneeded surgeries. The FNA biopsy is widely recognized as a minimally invasive yet highly precise method for the pathological diagnosis of thyroid nodules. Its approach is broadly accepted and closely aligned with the guidelines for thyroid nodule management and treatment. Notably, the average false negative rate for thyroid nodule biopsy typically falls between 3 and 5% (20), although larger nodules may have a higher error rate.

Ultrasound remains the primary imaging technique for diagnosing TNs, enabling early detection, which is essential for effective management and positive outcomes. Key ultrasound parameters strongly predictive of malignancy include microcalcifications (characterized by punctate echo foci lacking a posterior shadow), large calcifications, shapes varying from tall to wide, irregular and needle-like boundaries, and pronounced hypoechogenicity (21). The ultrasound findings indicate the existence of benign nodules, encompassing significant cystic elements, hyperechoic solid nodules, clear comet tail artifacts in punctate foci, and a spongy texture. Large cystic or cavernous nodules are inherently benign in nature (22, 23). Ultrasonography is the simplest and most efficient method for monitoring common non-functional benign thyroid nodules. The key observation goals encompass evaluating whether the nodule volume has notably increased, identifying malignant indicators or abnormal lymph nodes, and establishing a foundation to ascertain the necessity of FNA. Nevertheless, the sonographer’s assessment level of TNs is contingent upon their clinical experience. Notably, identifying the aforementioned malignant signs heavily relies on the sonographer’s expertise. Specifically, preoperative ultrasound diagnosis accuracy may be inadequate when performed by less experienced or primary care physicians. Consequently, it is imperative to reduce the reliance on ultrasound experience.

The necessity for a thyroid biopsy is typically influenced by various factors, including but not limited to the ultrasound risk assessment profile, the existence of compressive symptoms or clinical suspicion of high-risk thyroid cancer, patient life expectancy, comorbidity burden, surgical risk, individual risk factors for thyroid cancer, thyroid function level, local resources, medical expertise, and patient values and preferences (24).

TNs are predominantly benign, with malignant tumors comprising approximately 5–10% of cases. Typically, the benign or malignant characteristics of the nodule can be discerned through a comprehensive assessment involving patient history, presenting symptoms, clinical signs, ultrasonography, FNA, laboratory tests, and genetic screenings. Thyroid cancer ranks as the ninth most prevalent cancer globally (25) and is a leading endocrine malignancy. Over the past three decades, the occurrence of thyroid cancer has escalated across numerous populations, wherein the rate of incidence among women is triple that of men (26). The signs of thyroid cancer typically include an enlarged thyroid gland or the presence of nodules. These nodules are often irregularly shaped, fixed, and adhere to adjacent tissues. They gradually increase in size, have a hard texture, and possess indistinct boundaries. Initially, they may move up and down during swallowing but become immobilized in later stages. If accompanied by lymph node metastasis in the neck, the lymph nodes become palpable and enlarged. Compression or invasion of the sympathetic nerve may lead to Horner’s syndrome. The WHO’s 2022 classification of thyroid tumors clearly categorizes thyroid tumors originating from follicular cells into benign, low-risk, and malignant groups (27). Thyroid cancer is categorized based on tumor origin and differentiation into several subtypes: papillary thyroid carcinoma (PTC), follicular thyroid carcinoma (FTC), medullary thyroid carcinoma (MTC), poorly differentiated thyroid carcinoma (PDTC), and anaplastic thyroid cancer (ATC). PTC stands as the most prevalent, representing approximately 90% of all thyroid cancers, and both PTC and FTC are collectively referred to as differentiated thyroid carcinoma (DTC). PTC commonly presents with regional lymph node metastasis, often appearing as multicentric thyroid nodules. Conversely, FTC demonstrates a higher likelihood of developing distant organ metastases compared to regional lymph node spread. While typical PTC carries a favorable prognosis, its variants (such as hypercellular, hoof-and-nail, solid, and columnar) display a more aggressive clinical course (28).

In the realm of managing TNs, more than 90% of the nodules are identified as benign and asymptomatic through ultrasonographic or cytological assessments conducted after initial screenings. These patients usually display regular thyroid function and do not require any particular therapeutic intervention (29). Long-term follow-up is generally recommended to detect any malignancies that may have been missed during the initial evaluation, as well as to rule out the potential development of compressive symptoms or malignant nodules, which can result in hyperthyroidism (30). In the event of changes in thyroid hormone secretion, it becomes crucial to conduct a further evaluation to determine the severity of hypothyroidism or hyperthyroidism. Surgical intervention stands as the traditional form of treatment for TNs, particularly when their growth leads to neck discomfort, stress-related symptoms, and cosmetic concerns that detract from the quality of life. Nevertheless, preserving thyroid function during these procedures remains paramount (31). If a nodule is suspected to be malignant, active surveillance may be considered a viable alternative to immediate surgery for carefully selected patients with low-risk malignancies. However, it is imperative to further ascertain the nature of the nodule and devise a treatment plan accordingly. In these individuals, PTCs less than 10–15 mm were observed, along with no indications of metastatic spread, suspicion of extrathyroidal expansion, or cytological features associated with tumor aggressiveness (32).

Ultrasonography is the recommended follow-up method for TNs. The frequency and interval of follow-up are primarily determined by the malignancy risk, which is assessed through suspicious ultrasound signs of nodules and FNA cytological findings. According to several current guidelines, follow-up surveillance is adequate for the majority of benign TNs and those that do not meet the FNA criteria, with no specific treatment required. The 2015 American Thyroid Association (ATA) guidelines (18) offer the following recommendations for FNA nodules initially considered inappropriate: Nodules with ultrasound results indicating a high level of suspicion for malignancy should undergo repeat ultrasound examinations every 6–12 months. On the other hand, nodules with low to moderate suspicion of malignancy should have repeat ultrasounds every 12–24 months. For nodules with a maximum diameter exceeding 1 cm, minimal suspicion of malignancy (encompassing cavernous nodules), and uncomplicated cystic nodules, the recommended interval for repeat ultrasound examination should be longer than 2 years. In contrast, routine ultrasound examination is not necessary for nodules with a maximum diameter of 1 cm or less, minimal suspicion of malignancy (including cavernous nodules), and purely cystic nodules. In the event of benign cytological findings, it is advisable to categorize the patient into a risk stratification group based on the features observed in the cytological images. For cytologically benign nodules, risk stratification should be conducted based on ultrasound imaging features to ascertain the appropriate follow-up frequency. It is worth noting that the nodules’ nature, including those reported as Bethesda III, IV, and V, remains undetermined. Nodules classified as V carry a higher malignancy risk, often aligning with treatment and monitoring protocols for malignant nodules. When considering malignant nodule cytology results, there is widespread consensus among leading domestic and international guidelines, recommending surgical intervention. Therefore, surgical treatment is advised.

In recent years, the emergence of advanced image information mining technology has sparked a renewed interest in deep learning for imageomics analysis. This approach utilizes many features to build predictive models aimed at diagnosis, prognosis, and treatment planning. Histological evaluations have shown their proficiency in distinguishing between benign and malignant TNs, achieving an impressive area under the receiver operating characteristic curve of 0.97 in a study involving subjects (33). The applications of deep learning are extensive and varied, covering the detection, diagnosis, and treatment of TNs in various fields. One such application is ultrasound image analysis (34), which includes the automated identification of TNs and the prediction of nodule categorization and malignancy potential. Additional imaging techniques, such as CT, MRI, and various others, can aid in the diagnosis (35). Multimodal data fusion; guidance for puncture biopsies (36); surgical planning and navigation (37); telemedicine and diagnostic assistance (38); analysis of pathology images; and personalized treatment plans, among others. The application of deep learning has shown significant potential in detecting and diagnosing TNs, not only improving diagnostic efficiency and accuracy but also paving the way for personalized treatment and intraoperative navigation. With the accumulation of data and advancements in algorithms, the use of deep learning in managing TNs will expand and deepen.

### 4.3 Limitations

First, the literature search was confined to the Web of Science databases, potentially leading to variations in results if alternative databases were employed. Additionally, newly published articles of exceptional quality frequently do not have enough time to accrue citations, which prevents them from appearing on the list of the top 100 most-cited articles, potentially resulting in citation bias. Various innovative diagnostic and treatment methods may have been overlooked. Finally, it should be noted that our study did not include high-quality research conducted in languages other than English. It is anticipated that future studies will encompass a broader range of emerging high-quality articles by incorporating a wider array of databases.

## 5 Conclusion

In this comprehensive study, we conducted a rigorous bibliometric analysis of the 100 most frequently cited articles in the domain of TNs, leveraging an array of advanced software tools. These articles, from 2003 to 2021, exhibit a notable concentration of publications and citations originating from the United States. Harvard University emerges as the foremost contributor to this scholarly corpus, with an impressive 605 researchers publishing across 45 journals. The primary research topics center around “fine needle aspiration” and “carcinoma,” while “deep learning” emerges as a prominent area of future exploration.

By analyzing key terms, we identified the following trends in TN research: First, diagnostic technology is evolving toward greater diversity and precision. Ultrasound is the primary diagnostic tool for TNs, providing non-invasive, convenient, and exceptionally sensitive capabilities. The introduction of multimodal ultrasound technology, specifically ultrasonic elastography and ultra-microvascular imaging, has significantly improved diagnostic efficiency, reducing the need for unnecessary biopsies. FNA biopsy, although essential in TN diagnosis, can be affected by numerous variables, potentially compromising accuracy. In recent years, diagnostic accuracy has been significantly improved by integrating FNA with molecular testing, such as BRAF mutation analysis. It is crucial to prioritize the identification and treatment of malignant lesions, while assessing the risk of malignancy in TNs through imaging, molecular markers, and other advanced methods. For example, in cases of low-grade malignancies, such as papillary thyroid microcarcinoma, minimally invasive techniques such as thermal ablation are actively being researched and implemented, and initial studies have shown promising results in terms of safety and effectiveness. Additionally, the clinical approach to managing TNs, including diagnosis and follow-up care, is gradually becoming more standardized and personalized. Taking into account the specific nodule traits and individual patient factors, personalized treatment plans have become increasingly popular. The diagnosis and management of TNs necessitate a collaborative, multidisciplinary approach that encompasses endocrinology, ultrasound imaging, pathology, surgery, and several other specialties. This collaborative framework ensures a deeper evaluation and tailored treatment. Future advancements may involve creating a holistic evaluation system that integrates multi-faceted data such as imaging results, molecular markers, and clinical manifestations. This would improve the precision of malignancy risk predictions related to nodules.

Studies are increasingly incorporating ultrasound images with extra clinical data such as patient age, gender, medical history, and other factors to improve diagnostic precision. Some studies propose using both transverse and longitudinal ultrasound image perspectives of TNs, along with integrated learning technology, to classify benign and malignant nodules. This comprehensive approach employs independent predictors from multiple viewpoints, providing richer information and ultimately enhancing diagnostic accuracy. Researchers are continuously refining and advancing the architecture of deep learning models to optimize their performance. Beyond merely diagnosing benign and malignant TNs, recent investigations are probing how these models can aid in subsequent treatment decisions. Presently, diagnosing TNs involves a degree of subjectivity, leading to inconsistencies in the interpretation of ultrasound images among doctors and consequently a significant misdiagnosis rate. By processing massive amounts of image data, deep learning models objectively identify nodule characteristics, enhancing diagnostic accuracy and reducing unnecessary biopsies. Additionally, diagnosing TNs requires significant time and effort from doctors analyzing ultrasound images. Deep learning models rapidly analyze and categorize these images, providing preliminary diagnostic insights to physicians, thereby lightening their workload. Currently, disparities exist in the diagnostic process of TNs across various medical institutions due to the lack of unified standards. Implementing a deep learning model can standardize the diagnostic process, providing clinicians with consistent outcomes and improving diagnostic standardization. Future research could focus on developing a deep learning-assisted diagnostic model specifically for TN ultrasound images, aiming to enhance diagnostic precision and efficiency. Alternatively, multicenter clinical trials could validate the practical utility of deep learning in differentiating benign from malignant TNs in clinical settings. Other potential research directions include using deep learning algorithms to untangle the complex relationships between TN gene data and clinical features or creating a TN risk assessment system that integrates multiple clinical indicators (e.g., age, gender, and family history) and imaging characteristics to predict potential risks.

After analyzing the content of this article, it becomes apparent that TN research is advancing toward a more accurate, standardized, and tailored methodology. Furthermore, the domain is vigorously exploring the utilization of new technologies and enhancing existing diagnostic and treatment processes.

## Data Availability

The original contributions presented in the study are included in the article/supplementary material, further inquiries can be directed to the corresponding author/s.
